# The Glucocorticoid Receptor Gene (*NR3C1*) 9β SNP Is Associated with Posttraumatic Stress Disorder

**DOI:** 10.3390/healthcare9020173

**Published:** 2021-02-05

**Authors:** Ivone Castro-Vale, Cecília Durães, Elisabeth F. C. van Rossum, Sabine M. Staufenbiel, Milton Severo, Manuel C. Lemos, Davide Carvalho

**Affiliations:** 1 Medical Psychology Unit, Department of Clinical Neurosciences and Mental Health, Faculty of Medicine, University of Porto, 4200-319 Porto, Portugal; 2 i3S—Instituto de Investigação e Inovação em Saúde (Institute for Research and Innovation in Health), Universidade do Porto, 4200-135 Porto, Portugal; cduraes@ipatimup.pt (C.D.); davideccarvalho@gmail.com (D.C.); 3 Ipatimup—Institute of Molecular Pathology and Immunology of the University of Porto, 4200-135 Porto, Portugal; 4 Erasmus MC, Division of Endocrinology, Department of Internal Medicine, University Medical Center Rotterdam, 3015 CE Rotterdam, The Netherlands; e.vanrossum@erasmusmc.nl (E.F.C.v.R.); sabinestaufenbiel@web.de (S.M.S.); 5 Department of Clinical Epidemiology, Predictive Medicine and Public Health, Faculty of Medicine, University of Porto, 4200-319 Porto, Portugal; milton@med.up.pt; 6 Department of Medical Education and Simulation, Faculty of Medicine, University of Porto, 4200-319 Porto, Portugal; 7 CICS-UBI—Health Sciences Research Centre, University of Beira Interior, 6200-506 Covilhã, Portugal; mclemos@fcsaude.ubi.pt; 8 Department of Endocrinology, Diabetes and Metabolism, Centro Hospitalar S. João, Faculty of Medicine, University of Porto, 4200-319 Porto, Portugal

**Keywords:** posttraumatic stress disorder, genetics, glucocorticoid receptor, *NR3C1*, single nucleotide polymorphism, war veterans, hair cortisol

## Abstract

Posttraumatic stress disorder (PTSD) has been associated with glucocorticoid (GC) hypersensitivity. Although genetic factors account for 30–46% of the variance in PTSD, no associations have been found between single nucleotide polymorphisms (SNPs) of the GC receptor (GR) gene (*NR3C1*) and risk for this disorder. We studied the association of five SNPs in the GR gene (rs10052957, rs6189/rs6190, rs6195, rs41423247, and rs6198) and haplotypes with PTSD, in a group of Portuguese male war veterans (33 with lifetime PTSD, 28 without). To determine whether the 9β SNP (rs6198) was associated with chronically altered cortisol levels, we evaluated hair cortisol concentrations (HCC) in a sample of 69 veterans’ offspring. The 9β variant (G allele) was significantly associated with lifetime PTSD under a dominant model of inheritance. The 9β variant was also significantly associated with severity of current PTSD symptoms. The haplotype analysis revealed an association between a common haplotype comprising the 9β risk allele and lifetime PTSD. Carriers of the 9β risk allele had significantly lower HCC than non-carriers. We found the 9β risk allele and a haplotype comprising the 9β risk allele of the GR gene to be associated with PTSD in veterans. This 9β risk allele was also associated with lower HCC in their offspring.

## 1. Introduction

Posttraumatic stress disorder (PTSD) has been associated with altered regulation of the hypothalamic pituitary adrenal (HPA) axis, more specifically with increased glucocorticoid (GC) sensitivity [1,2]. This has been shown by means of dexamethasone suppression tests and an increased glucocorticoid receptor (GR) number [2,3]. However, lower levels of cortisol have mainly been found in specific populations and at specific moments of the day [4]. Measurement of hair cortisol concentrations (HCC) is a relatively recent and reliable methodology reflecting the systemic effects of cortisol that allow assessment of HPA axis regulation in the previous months, depending on the length of the hair segment [5]. Thus, HCC represent cumulative cortisol levels over prolonged periods of time providing insight into chronic HPA axis dysregulation [6]. Studies have found lower HCC to be associated with PTSD in female [7] but not in male [8] study cohorts. HCC have also been influenced by time since trauma, type of trauma, childhood adversities, and hair manipulations [9,10]. To our knowledge, there are no studies investigating GR gene (*NR3C1*) single nucleotide polymorphisms (SNPs) associations with HCC.

Since genetic factors have been estimated to account for 30–46% of the variance in PTSD [11,12], SNPs of the GR gene that are associated with altered sensitivity to GCs have been studied for this disorder. The SNPs N363S (rs6195; alias rs56149945) and B*cl*I (rs41423247) have been associated with increased sensitivity to GCs, while ER22/23EK (rs6189/rs6190) and 9β (rs6198) have been associated with a relative GC resistance. The *Tth111*I (rs10052957) SNP only showed clinical associations when simultaneously present with ER22/23EK [13]. Although no main effects have been found for any of the GR gene SNPs in PTSD, severity of PTSD and basal cortisol levels was negatively correlated in a subset of war veterans with PTSD homozygous for the B*cl*I SNP. In the same subset of veterans, a tendency to increased response to a test of peripheral GC sensitivity that correlated with higher PTSD symptoms was also found [14]. In a study of cardiac surgery patients, homozygous carriers of the B*cl*I SNP showed significantly lower preoperative plasma cortisol levels and more traumatic memories after six months [15]. Van Zuiden et al. [16] found a significant interaction of the haplotype comprising the B*cl*I SNP and childhood trauma on the pre-deployment GR number. In their study, childhood trauma and pre-deployment high GR number both predicted subsequent development of high levels of PTSD symptoms. The higher GR number in the PTSD group was maintained after one and six months [3].

The aim of this study was to investigate the associations of SNPs in the GR gene (*Tth111*I, ER22/23EK, N363S, *Bcl*I, and 9β) and haplotypes with lifetime PTSD in war veterans.

Since we found associations between the SNP 9β and lifetime and current PTSD, we performed an analysis aimed at determining whether carrying 9β risk allele was associated with HPA axis function measuring HCC.

## 2. Material and Methods

### 2.1. Enrolment of Participants

The present cross-sectional research is part of a larger study on the neurobiological inheritance of PTSD (e.g., [17]). This study was approved by the Ethics Committee for Health of Centro Hospitalar São João/Faculty of Medicine of the University of Porto (approval number: CES-138/08). After having received a written and verbal description of the study, all participants who agreed to participate gave their written informed consent. We used two methods to select participants: 75.4% were from an outpatient clinic of the Portuguese Disabled Veterans Association (ADFA), and 24.6% were from three lists of veterans’ companies from the Portuguese colonial wars. A total of 61 male, Caucasian veterans agreed to participate. In this sample 36.1% had been recruited to Angola, 31.1% to Mozambique, and 32.8% to Portuguese Guinea (currently Guinea-Bissau) during the war. Women did not participate in the Portuguese colonial wars as combatants.

Participants were included if they fulfilled the war-related Diagnostic and Statistical Manual of Mental Disorders (DSM-IV) criterion A for PTSD and had children. The general exclusion criteria for participants were the presence of neurologic, infectious, or any active medical illness, and any DSM-IV psychotic, bipolar, or cognitive disorders. Participants with PTSD were also excluded if they had current substance-related disorders. Specific control group exclusion criteria included previous PTSD diagnosis and any current psychiatric disorder.

For the analysis of HCC, a sample of the veterans’ offspring was used to reduce confounding variables. Seventy-two offspring of 45 veterans agreed to participate. In the case of two offspring (both from the group of fathers with lifetime PTSD), HCC could not be measured, because the concentrations were outside the equipment detection limits. One offspring (whose father did not have lifetime PTSD) had taken oral glucocorticoids during the previous three months and was therefore excluded.

### 2.2. Psychosocial Measures

Sociodemographic data and clinical history were collected. Socioeconomic status (SES) was measured using the Graffar Index. The Graffar Index classifies subjects into five classes, score one being the highest, and five the lowest.

The Portuguese version of the Clinician-Administered PTSD Scale (CAPS) was used to characterize the veterans in relation to PTSD diagnosis. Lifetime PTSD was considered if participants had DSM-IV criteria according to Blake et al.’s rule [18] (frequency ≥ 1 and intensity ≥ 2) and a total CAPS score of 50 or more. The CAPS score was also used as a continuous measure of PTSD symptoms. In this case the highest lifetime PTSD score measured was considered. Veterans were also grouped according to those who never had PTSD, veterans who had past PTSD, and veterans who have current PTSD with the previously mentioned criteria and rule. Traumatic events (TEs) were assessed with the CAPS Life Events Checklist (LEC), and subsequently checked for the DSM-IV A2 criterion, following the CAPS procedure. In order to determine participants’ eligibility for the study, current and lifetime psychiatric disorders were investigated with the Structured Clinical Interview for DSM-IV axis I (SCID-I) disorders (Portuguese Version Ângela Maia. Unpublished manuscript, Department of Psychology, University of Minho, Braga, Portugal).

To study the association of HCC with the SNP 9β in the veterans’ offspring the following variables were also investigated as they can influence HCC.

The number of TEs experienced was quantified using the CAPS LEC, as described for their fathers. The offspring also filled out a questionnaire concerning hair conditions. Childhood trauma was investigated using the Portuguese version of the Childhood Trauma Questionnaire-Short Form (CTQ-SF) [19,20]. The CTQ-SF is a self-reporting measure, which has been validated in a Portuguese non-clinical sample with a Cronbach alpha for the total score of 0.84 [20] and 0.80 in our sample of offspring of the veterans. The total CTQ-SF score used in the present study provides a general childhood adversity score—and not just for TEs. The questionnaire contains 28 items, which enquire about specific maltreatment experiences. Items are classified into a five-point Likert scale, according to the frequency of exposure to that specific experience.

### 2.3. GR gene SNPs Genotyping

DNA was extracted from frozen oral mucosa scrape using the MagCore 401 DNA extraction kit (RBC Bioscience, New Taipei City, Taiwan), according to the manufacturer’s guidelines.

Five polymorphisms in the GR gene *NR3C1* (*Tth111I*, ER22/23EK, N363S, *Bcl*I, and 9β) were genotyped, using TaqMan allelic discrimination assays (Applied Biosystems, Carlsbad, CA, USA), following protocols described by the supplier. Genotyping results were analyzed using the sequence detection system 2.2 software (Applied Biosystems, Carlsbad, CA, USA).

### 2.4. Hair Cortisol Measurements

Approximately 100 strands of hair from the posterior vertex of the scalp were cut off as close to the scalp as possible. The hair was taped to a piece of paper and the scalp end was marked. The samples were stored at room temperature and protected from light exposure until the analyses were carried out.

Cortisol concentrations of hair segments from the offspring were analyzed by liquid chromatography tandem-mass spectrometry (LC-MS/MS). The most proximal 1–3 cm of hair were used for analysis, as available. Based on the hair growth rate of 1 cm per month, these hair samples reflected the cumulative cortisol secretion of the previous one to three months. The sample preparation and analysis has been extensively described elsewhere [21]. Briefly, the samples were cut into 1 cm segments, weighed, and then washed with 2 mL isopropanol for two minutes. The extraction of cortisol was achieved by overnight incubation with 1.4 mL methanol and the presence of 100 μL internal standard (cortisol-d4) for 18 h, at 25 °C and low agitation.

After extraction, the methanol was transferred to a glass vial and then centrifuged at 4500 rpm, at 4 °C for 5 min. One mL of clear supernatant was then transferred into a new glass vial and this was evaporated under a soft stream of nitrogen at 50 °C. The dried samples were then reconstituted in 1 mL 2% methanol, and vortexed at 2000 rpm for 1 min. The samples were further cleaned by solid phase extraction. Samples were evaporated at 50 °C under a constant flow of nitrogen. Before injection, the dry residue was re-suspended in 30% methanol. Steroid peak integrations were reviewed and were manually integrated by two independent persons when automated peak integration occurred incorrectly, or peaks were partially integrated.

Five offspring had hair strands of less than 3 cm length, but at least 1 cm, and were therefore included. No differences in HCC, expressed in pg per mg of hair, were found between participants with 3 cm when compared with those with at least 1 cm.

### 2.5. Statistical Analysis

Sociodemographic characteristics differences between the groups of veterans were analyzed with t tests for continuous variables, and chi-square or Fisher’s exact tests for categorical variables.

Association of the GR gene SNPs (conferring GC resistance or hypersensitivity) with lifetime PTSD (no PTSD versus PTSD) was assessed using an unconditional logistic regression model. Crude odd ratios (OR) with 95% confidence intervals (CI) and *p-*values were calculated. Mann–Whitney tests were used to compare the median of current PTSD symptom severity between carriers and non-carriers of any risk allele of SNPs conferring resistance or hypersensitivity categories, and also between carriers and non-carriers of the 9β SNP risk allele categories. Association of the GR gene SNPs (conferring GC resistance or hypersensitivity) with PTSD classified in three ordered categories (veterans who had never had PTSD, veterans who had had PTSD in the past, and veterans who still had PTSD at the moment of assessment) was assessed using an unconditional ordinal regression model. Proportional odds with 95% CI and *p-*values were calculated.

For the analysis of the offspring samples, sociodemographic, psychometric, and hair characteristic associations with ln HCC (pg/mg), as well as the association between carrier state of SNP 9β (rs6198) variant and ln HCC, were analyzed using mixed effects model with random intercept by family to account the dependency between the observations of the same family.

The *p*-values presented are two-sided and a *p* < 0.05 was considered statistically significant. Genotype associations were analyzed using SPSS 23 (IBM, Armonk, NY, USA), and haplotype associations were analyzed using the package “SNPassoc” implemented in R (minimum haplotype frequency > 0.01) (open source project: https://www.R-project.org/ (accessed on 4 February 2021)). We did not adjust for multiple testing since all SNPs are located in the same gene, and mutually exclusive, thus not completely independent.

## 3. Results

### 3.1. Associations of Genetic Variation in the GR Gene with Lifetime PTSD

Of the 61 veterans enrolled (mean age, standard deviation (SD): 65.2, 3.4), 33 presented lifetime PTSD (64.8, 3.4) and 28 did not (65.8, 3.3). Ninety-three percent were married, 13.1% were classified in Class 2 of the Graffar Index, 62.3% in Class 3, and 24.6% in Class 4. There were no veterans classified in either Class 1 or 5 of the Graffar Index. Thirty percent of the veterans had a disability. Mean body mass index (BMI) was 27.7 kg/m^2^ (SD = 3.0). We observed no differences in the groups with and without lifetime PTSD, with respect to age, marital status, Graffar index, BMI, presence of disability, or the deployment region to which they were recruited.

The genotype frequencies of all SNPs were consistent with the Hardy–Weinberg equilibrium in the control group (except for the *Bcl*I, which was borderline (*p-*value = 0.048)). Table 1 shows the genotype frequencies and the OR, CI, and *p-*value calculated for the minor allele under the dominant model for the five SNPs. Only the 9β SNP allele G (dominant model) reached significance with an OR (CI) of 3.58 (1.09–11.80) and *p-*value = 0.036.

Carrying at least one of the risk alleles of SNPs previously associated with GC resistance (ER22/23EK and 9β) was associated with lifetime PTSD at a trend-level (OR (CI) = 3.29 (0.98–11.03); *p-*value = 0.053, not shown in the table). No associations between any SNP associated with GR hypersensitivity (N363S and *Bcl*I) and lifetime PTSD were observed.

Table 2 shows the genotype frequencies and the OR, CI, and *p-*value calculated for the minor allele under the dominant model for the five SNPs according to the veterans’ present PTSD situation (never had PTSD, had PTSD in the past, and still had PTSD at the moment of assessment). Only the 9β SNP allele G (dominant model) reached significance with an OR (CI) of 0.31 (0.11–0.90) and *p-*value = 0.030. The relative frequency of the 9β risk allele was higher in the group who still had PTSD compared with the group that only had PTSD in the past, and the group that never had PTSD. Carrying at least one of the risk alleles of the SNPs previously associated with GC resistance (ER22/23EK and 9β) was also associated with the veterans’ present PTSD situation, with an OR (CI) of 0.32 (0.11–0.92), *p-*value = 0.035.

Subsequent analyses considering veterans having at least one of the 9β or ER22/23EK risk alleles (median = 38.00, interquartile range (IQR) = 59) had significantly more current PTSD symptoms than those not carrying any risk allele (median = 0.00, IQR = 26), *p-*value = 0.011. Moreover, veterans carrying at least one 9β risk allele (median = 38.00; IQR = 59) had significantly more current PTSD symptoms than those not carrying any risk allele (median = 0.00, IQR = 26), *p-*value = 0.010.

Haplotype analysis (Table 3) showed that the common haplotype TGACG (Haplotype 4) carrying the 9β risk allele (20.3%) was significantly associated with having lifetime PTSD (OR (CI) = 19.03 (1.59–227.92); *p-*value = 0.020; order of SNPs is indicated on Table 3). The association was retained after adjustment by each SNP in the haplotype.

### 3.2. Association of HCC with the GR Gene SNP 9β

The final offspring samples of 69 participants (mean age 35.4 years (SD = 4.8), 59.4% females) had a mean BMI of 26.4 kg/m^2^ (SD = 4.4). There were no fixed linear regression coefficient statistical associations between age, BMI, gender, SES, hair characteristics, smoking, daily alcohol consumption, MDD, total CTQ-SF score, total number of TEs, use of regular medication, locally applied GC, psychoactive medication, or hormonal contraceptives in women, and HCC. We also did not find an association between the offsprings’ fathers’ lifetime PTSD diagnosis and HCC.

The fixed linear regression coefficient showed a significant positive association between those offspring carrying the SNP 9β risk allele and HCC (pg/mg) (Exp (β) = 0.75; CI: 0.56, 0.99, *p-*value = 0.042).

## 4. Discussion

We studied the relationships between GR gene polymorphisms, known to be associated with altered GC sensitivity, and PTSD in war veterans. In addition, we studied the relation of GR gene SNP 9β variation with chronic cortisol exposure, as measured with hair analysis.

We observed that the 9β G risk allele was associated with lifetime PTSD and with current PTSD symptoms and current PTSD diagnosis. Since this is the first study reporting this association, it should be replicated in a different population. The presence of at least one variant allele of the SNPs ER22/23EK and 9β, previously associated with GC resistance, was also associated with current PTSD symptoms and current PTSD diagnosis. However, these associations are mainly explained by the presence of the 9β G risk allele. Moreover, we found a negative association between the 9β risk allele G and HCC in veterans’ offspring, which supports the GC hypersensitivity that has been previously reported in patients with PTSD. Our results also favor the consideration that the 9β risk allele might contribute to PTSD chronicity, as it was also associated with current PTSD and current PTSD symptoms.

The 9β SNP encodes mRNA for the hGRβ isoform, which potentially functions as a dominant negative inhibitor of hGRα activity by increasing mRNA stability and receptor protein expression [22,23]. The 9β polymorphism has been related to GC resistance in men shown as by higher adrenocorticotropic hormone (ACTH) levels and cortisol awakening response peak levels after dexamethasone and the highest ACTH and serum cortisol response to psychosocial stress in males [24]. At a functional level, van den Akker, et al. [25] using human bio-assays, showed decreased transrepression of the GR, which may lead to a pro-inflammatory state. This SNP has been shown to confer increased stability to the GRβ isoform, which in turn exerts antagonistic action on the classic GRα [22]. It is suggested that increased expression levels of the GRβ splice variant results in cellular resistance to GCs. However, PTSD has been associated with GC hypersensitivity, mainly through findings of increased suppression of cortisol in the dexamethasone suppression test [2], however this is controversial. Some recent meta-analytic studies have found that GC hypersensitivity is related to trauma exposure and is independent of PTSD development (e.g., [4]). On the other hand, tissue specific SNP functionality has been found for *Bcl*I [26] and this might apply to the other SNPs as well, although the expression of the GRβ splice variant is low for cells other than immune cells [27]. In fact, the decreased transrepression associated with the 9β SNP risk allele is consistent with a more active immune system [28], which has also been found in PTSD patients [29]. Increased risk for cardiovascular disease has also been associated with the 9β SNP risk allele [30], which is a common comorbidity among patients with PTSD [31]. Studies of the 9β SNP risk allele are still controversial with regard to GR resistance, as for example this SNP has been associated with a protective effect for affective disorders [32]. Affective disorders have been consistently associated with GR resistance [33]. On the other hand, meta-analytic studies have found PTSD to be associated with lower levels of cortisol in specific populations [4], particularly with females [34]. However, these meta-analyses comprised cortisol output and post-dexamethasone cortisol levels but no long-term cortisol levels as can be measured using hair analysis. Moreover, in the case of HCC, some studies seem to support the idea that PTSD could be associated with lower cortisol in female [7,35] but not in male [8] samples. PTSD is a complex disorder with multiple risks factors interplaying. This may, in part, explain the finding in this study of a SNP previously implicated in GR resistance to be associated with PTSD. As our sample of veterans was exclusively male, this could be one of the factors contributing to the results. Another explanation could be the occurrence of a gene–environment interaction not addressed in this study, reported to occur with other SNPs in the GR gene and other genes influencing the HPA axis function, such as the *FKBP5* [16,36]. Being a carrier of the 9β SNP risk allele could interact with war traumatic load or even with childhood adversities to increase the risk to develop PTSD.

Furthermore, the association of the 9β SNP risk allele with GR resistance is not corroborated with further results from another sampling group in this study showing an association of this SNP allele with lower HCC, which may be associated with GR hypersensitivity. HCC is a reliable methodology reflecting the systemic effects of cortisol that allow insight into chronic HPA axis dysregulation [6]. GCs have an important role in the processing of emotional memories of trauma [37]. Enhanced GC signaling during stress has been shown to increase emotional memories. Moreover, lower levels of GCs may result in facilitated retrieval of traumatic information and long-term failure to extinguish traumatic memories. Together these effects constitute one hypothesis for PTSD pathophysiology [38].

Haplotype analysis showed an association between Haplotype 4 and lifetime PTSD. This haplotype contains the 9β SNP risk allele reported in the single SNP analysis. This finding corroborates the association of carrying the 9β risk allele with lifetime PTSD. Although this haplotype also includes the *Tth111*I risk allele, this SNP has only been shown to be functional when associated with ER22/23EK [13]. To our knowledge, no other studies investigated the association of GR gene haplotypes with PTSD.

This is the first study to investigate the association between the 9β and *Tth111*I SNPs in the GR gene and PTSD. Other studies have addressed other functional GR gene SNPs—ER22/23EK, N363S, and *Bcl*I [14], or have only studied PTSD symptoms [15,16], but no main effects have been found between these SNPs and PTSD. To our knowledge, this is also the first study to investigate the association between the 9β SNP association with HCC. Since altered function of the GR has been associated with PTSD in several studies [1] and GR gene SNPs have been associated with altered function of the HPA axis, and particularly of the GR [13,24,25], there is a high a priori probability that SNPs of this gene are associated with PTSD. Furthermore, these SNPs are all located in the same gene having a high probability of being inherited together. This would allow sparing correction for multiple testing, as the tests are not completely independent. Furthermore, a recent meta-analysis found SNPs in GR and FKBP5 genes to be significantly associated with PTSD [39].

Strengths and limitations: Concerning the study of the association of the GR gene SNPs with lifetime PTSD, veterans were all male and Caucasian, and therefore the results may not be generalized to other populations. This may, however, represent a strength of the study, as variables such as age, gender, and ethnicity did not confound the results. The results cannot be generalized to PTSD related to TEs other than war-related ones. Another limitation is the veterans’ small sample size, which could hide small SNPs effects. We cannot rule out that our findings result from type I errors, however, on the other hand, possible type II errors are reduced. Our use of CAPS, which is the gold standard instrument used to diagnose and measure PTSD, is certainly a strength of this research. Concerning the negative association of HCC with the SNP 9β found in a distinct, young, relatively healthy, and homogeneous sample with regard to their fathers all having had experienced war-related TEs was also a strength.

## 5. Conclusions

In summary, we found an association between the GR gene 9β SNP risk allele and lifetime and current PTSD, and current PTSD symptoms in the sample of veterans from Portugal. *Tth111*I, ER22/23EK, *Bcl*I, and N363S SNPs alone were not associated with increased risk of developing PTSD. The common Haplotype 4 (also comprising the 9β risk allele) was also associated with lifetime PTSD. Although we have to interpret the results with caution, as they need replication in a larger cohort, our findings suggest the 9β risk allele G and the haplotype comprising the 9β risk allele of the GR gene are genetic susceptibility factors for the development and maintenance of lifetime PTSD in war veterans. The association we found, in the sample of offspring, between 9β risk allele carriers and lower HCC, supports the association of this allele with HPA axis dysregulation, namely GR hypersensitivity, which has been found in patients with PTSD.

## Figures and Tables

**Table 1:** Genotypic frequencies and association of the glucocorticoid receptor (GR) gene genetic variants 9β, *Bcl*I, N363S, ER22/23EK, and *Tth111*I with lifetime PTSD in war veterans ^a^.

Locus/Genotype	No PTSD *n* (%)	PTSD *n* (%)	OR (95% CI)	*p-*Value
***Tth111*I (rs10052957)**	*n* = 28	*n* = 32		
CC	15 (53.6)	14 (43.8)		
CT	11 (39.3)	13 (40.6)		
TT	2 (7.1)	5 (15.6)		
T *carrier* vs. CC ^b^	13 (46.4)/15 (53.6)	18 (56.3/43.8)	1.48 (0.54–4.11)	0.448
**ER22/23EK (rs6189/rs6190)** ^c^	*n* = 24	*n* = 31		
GG/GG	22 (91.7)	29 (93.5)		
GA/GA	2 (8.3)	2 (6.5)		
AA/AA	0	0		
A/A *carrier* vs. GG/GG ^b^	2 (8.3)/22 (91.7)	2 (6.5)/29 (93.5)	0.76 (0.10–5.82)	0.790
**N363S (rs6195)**	*n* = 28	*n* = 33		
AA	25 (89.3)	32 (97.0)		
AG	3 (10.7)	1 (3.0)		
GG	0	0		
G *carrier* vs. AA ^b^	3 (10.7)/25 (89.3)	1 (3.0)/31 (97.0)	0.26 (0.03–2.66)	0.266
***Bcl*I (rs41423247)**	*n* = 27	*n* = 32		
CC	9 (33.3)	14 (43.8)		
CG	17 (63.0)	13 (40.6)		
GG	1 (3.7)	5 (15.6)		
G *carrier* vs. CC ^b^	18 (66.7)/9 (33.3)	18 (56.3)/14 (43.8)	0.64 (0.22–1.86)	0.415
**9β (rs6198)**	*n* = 28	*n* = 32		
AA	23 (82.1)	18 (56.3)		
AG	5 (17.9)	12 (37.5)		
GG	0	2 (6.3)		
G *carrier* vs. AA ^b^	5 (17.9)/23 (82.1)	14 (43.8)/18 (56.3)	**3.58 (1.09–11.80)**	**0.036**

PTSD = Posttraumatic stress disorder; OR = crude odds ratio with 95% confidence intervals; CI = confidence interval. ^a^ The number of individuals genotyped for each single nucleotide polymorphism (SNP) differs according to their genotyping success. ^b^ Reference genotype. nc: not calculated (the frequency of the homozygous minor allele is zero). ^c^ These two SNPs are in tight linkage. Bold font indicates nominally significant results.

**Table 2:** Genotypic frequencies and association of the GR gene genetic variants 9β, *Bcl*I, N363S, ER22/23EK, and *Tth111*I with war veterans’ PTSD classified in three ordered categories ^a^.

Genotype	Veterans’ PTSD Situation (%)			Univariate Analysis	
	Never Had ^b^ *n* (%)	Had in the Past *n* (%)	Have Current PTSD *n* (%)	Odds (95% CI)	*p-*Value
**Tth111l** **(rs10052957)**					
CC	15 (53.6)	10 (47.6)	4 (36.4)		
CT	11 (39.3)	9 (42.9)	4 (36.4)		
TT	2 (7.1)	2 (9.5)	3 (27.3)		
T carrrier versus CC ^c^	13 (46.4)/15 (53.6)	11 (52.4)/10 (47.6)	7 (63.6)/4 (36.4)	0.64 (0.24–1.67)	0.357
**ER22/23K (rs6189/rs6190)**					
GG/GG	22 (91.7)	20 (95.2)	9 (90.0)		
GA/GA	2 (8.3)	1 (4.8)	1 (10.0)		
AA/AA	0	0	0		
A/A carrier versus GG/GG ^c^	2 (8.3)/22 (91.7)	1 (4.8)/20 (95.2)	1 (10.0)/9 (90.0)	1.06 (0.16–7.15)	0.954
**N363S (rs6195)**					
AA	25 (89.3)	21 (100.0)	11 (91.7)		
AG	3 (10.7)	0	1 (8.3)		
GG	0	0	0		
G carrier versus AA ^c^	3 (10.7)/25 (89.3)	0/21 (100.0)	1 (8.3)/11 (91.7)	2.82 (0.33–24.45)	0.346
***Bcl*I (rs41423247)**					
CC	9 (33.3)	8 (40.0)	6 (16.7)		
CG	17 (63.0)	9 (45.0)	4 (33.3)		
GG	1 (3.7)	3 (15.0)	2 (50.0)		
G carrier versus CC ^c^	18 (66.7)/9 (33.3)	12 (60.0)/8 (40.0)	6 (50.0)/6 (50.0)	1.62 (0.61–4.33)	0.333
**9β (rs6198)**					
AA	23 (82.1)	12 (60.0)	6 (50.0)		
AG	5 (17.9)	6 (30.0)	6 (50.0)		
GG	0	2 (10.0)	0		
G carrier versus AA ^c^	5 (17.9)/23 (82.1)	8 (40.0)/12 (60.0)	6 (50.0)/6 (50.0)	**0.31 (0.11–0.90)**	**0.030**
**GC Hypersensitivity (N363S_BclI)** ^d^					
Carrier versus non carrier ^c^	21 (75.0)/7 (25.0)	12 (60.0)/8 (40.0)	7 (58.3)/5 (41.7)	1.87(0.68–5.11)	0.224
**GC Resistance (ER22/23EK_9β** **) ^e^**					
Carrier versus non carrier ^c^	5 (20.0)/20 (80.0)	8 (40.0)/12 (60.0)	6 (54.5)/5 (45.5)	**0.32 (0.11–0.92)**	**0.035**

PTSD = Posttraumatic stress disorder. ^a^ The number of individuals genotyped for each SNP differs according to their genotyping success. ^b^ Never had: reference category for ordinal regression. ^c^ Reference genotype. ^d^ Carrier of at least one of the risk alleles of the SNPs previously associated with GC hypersensitivity (N363S or *Bcl*I). ^e^ Carrier of at least one of the risk alleles of the SNPs previously associated with GC resistance (ER22/23EK or 9β). nc: not calculated (the frequency of the homozygous minor allele is zero). Bold font indicates nominally significant results.

**Table 3:** GR gene haplotypes frequencies and association with lifetime PTSD in war veterans.

Haplotypes ^a^	Haplotypes no.	No PTSD	PTSD	OR	CI (95%)	*p*-Value
CGACA ^b^	Haplotype 1	0.445	0.385	1.00		
CGAGA	Haplotype 2	0.212	0.240	2.49	0.76–8.22	0.133
TGAGA	Haplotype 3	0.143	0.107	1.57	0.38–6.52	0.532
TGACG	Haplotype 4	0.127	**0.203**	**19.03**	**1.59–227.92**	**0.020**
TAACG	Haplotype 5	0.039	0.031	0.84	0.08–8.33	0.881
CGGCA	Haplotype 6	0.027	0.016	1.35	0.03–63.65	0.877

PTSD = Posttraumatic stress disorder. ^a^ Alleles are ordered according to the following SNPs (chromosome 5 DNA reverse strand): *Tth111*I (rs10052957) C/T, ER22/23EK (rs6189/rs6190) G/A, N363S (rs56149945) A/G, *Bcl*I (rs41423247) C/G, and 9β (rs6198) A/G; ^b^ The most frequent haplotype (CGACA) was used as a reference; associations with *p*-value < 0.05 are highlighted in bold.

## Data Availability

The datasets used and analyzed for this study are available from the corresponding author upon request.
